# Combating Biofilm by Targeting Its Formation and Dispersal Using Gallic Acid against Single and Multispecies Bacteria Causing Dental Plaque

**DOI:** 10.3390/pathogens10111486

**Published:** 2021-11-15

**Authors:** Aqel Albutti, Muhammad Shoaib Gul, Muhammad Faisal Siddiqui, Farhana Maqbool, Fazal Adnan, Ihsan Ullah, Ziaur Rahman, Sadia Qayyum, Muhammad Ajmal Shah, Muhammad Salman

**Affiliations:** 1 Department of Medical Biotechnology, College of Applied Medical Sciences, Qassim University, Buraydah 51452, Saudi Arabia; as.albutti@qu.edu.sa; 2 Department of Health and Biological Sciences, Abasyn University, Peshawar 25000, Pakistan; shoaibbangash@yahoo.com (M.S.G.); s.amazai@yahoo.com (M.S.); 3 Department of Microbiology, Hazara University, Mansehra 21120, Pakistan; fairy_es11@yahoo.com (F.M.); sadiahu83@gmail.com (S.Q.); 4 Atta ur Rahman School of Applied Biosciences, National University of Sciences & Technology, Islamabad 44000, Pakistan; fazliadnan@gmail.com; 5 Department of Biological Sciences, Faculty of Science, King Abdulaziz University, P.O. Box 80203, Jeddah 21589, Saudi Arabia; iullah@kau.edu.sa; 6 Department of Microbiology, Abdul Wali Khan University, Mardan 23200, Pakistan; zrahman@awkum.edu.pk; 7 Department of Pharmacognosy, Faculty of Pharmaceutical Sciences, Government College University, Faisalabad 38000, Pakistan; ajmalshah@gcuf.edu.pk

**Keywords:** biofilm control, dispersal effects, potential effect, gallic acid, biomass, EPS, dental plaque

## Abstract

Exploring biological agents to control biofilm is a vital alternative in combating pathogenic bacteria that cause dental plaque. This study was focused on antimicrobial, biofilm formation and biofilm dispersal efficacy of Gallic acid (GA) against bacteria, including *Proteus* spp., *Escherichia coli*, *Pseudomonas* spp., *Salmonella* spp., *Streptococcus mutans*, and *Staphylococcus aureus* and multispecies bacteria. Biofilm was qualitatively and quantitatively assessed by crystal violet assay, florescence microscopy (bacterial biomass (µm^2^), surface coverage (%)) and extracellular polymeric substances (EPS). It was exhibited that GA (1–200 mg/L) can reduce bacterial growth. However, higher concentrations (100–200 mg/L) markedly reduced (86%) bacterial growth and biofilm formation (85.5%), while GA did not exhibit any substantial dispersal effects on pre-formed biofilm. Further, GA (20–200 mg/L) exhibited 93.43% biomass reduction and 88.6% (*p* < 0.05) EPS (polysaccharide) reduction. Microscopic images were processed with BioImageL software. It was revealed that biomass surface coverage was reduced to 2% at 200 mg/L of GA and that 13,612 (µm^2^) biomass was present for control, while it was reduced to 894 (µm^2^) at 200 mg/L of GA. Thus, this data suggest that GA have antimicrobial and biofilm control potential against single and multispecies bacteria causing dental plaque.

## 1. Introduction

Dental plaque and caries are major oral diseases around the globe and are known to be caused by different microbes and food particles [1]. Many oral bacteria, such as *Streptococcus mutans*, colonize on the teeth surface and are capable to produce acid by fermentation of sucrose and fructose, causing destruction of teeth structure and dental caries [2,3]. The population of microbes embedded in a matrix that builds up immediately on the surfaces of teeth are known as biofilms or dental plaque [4].

Biofilm is the colonization of bacteria entrapped in the exopolymeric matrix formed by the microbial cells. This matrix of biofilm is usually known as glycocalyx, which is typically composed of lipids, lipopolysaccharides [5,6], proteinaceous substances, glycopeptides [7], complex polysaccharide matrix and some other substances that hold different microorganisms together [8].

An ordered series of events occur in the dental plaque formation. Immediately after cleaning the teeth, dental plaque forming bacteria adsorbs to the teeth surfaces. Many of them are obligately anaerobic, and highly specific bacteria, creating suitable conditions for altering surrounding the environment for colonization. Biofilm produced by bacteria is heterogeneous in composition with open fluid filled channels running through the plaque mass. The fluid channels act as a circulatory system, which enables the bacteria to absorb the nutrients and proliferate within the matrix. Finally, microorganisms of a diverse community form a thick biofilm [9]. For the development of diverse oral biofilm, the interaction of bacteria is also necessary [10].

The aggregation of constituents in the oral cavity comprises complex substances e.g., dietary products, saliva, teeth, and oral bacteria closely interacting to each other [11]. Food product and its constituent cause dental caries formation in two ways, i.e., inducing defects on the texture of the tooth by reducing the pH of oral cavity, and altering the normal flora of oral cavity. As a result, dental caries develops by caries-associated bacteria [12]. 

Oral diseases, such as gingivitis, periodontitis, dental caries, and plaque, are the major leading health problems for most people around the world [13]. It is now well recognized that some microbial species in oral cavity have a convincing connection with oral infections [3,14]. Studies have shown that dental plaque have more than 1000 strains of bacteria (50% of them are unknown) and oral diseases have a similar number of these bacterial strains [13]. *S. mutans* is one of the major bacterium believed to be highly responsible for oro-dental diseases. *S. mutans* is the most observed pathogen that has been comprehensively studied in dental plaque and dental caries [15,16].

The resistance of antibiotics against the bacterial biofilm have been identified. Different mechanisms are involved in resistance of antibiotics i.e., (i) the inactivation of antibiotics in the EPS or poor penetration of drugs, (ii) the inactive and altered metabolic state of microbes, (iii) the presister cells, which are present in biofilm, and (iv) uses of sublethal concentration of antibiotics itself and the activation of efflux pumps. Multiple factors are involved in resistance of biofilm and may vary in different organisms [17].

Meanwhile, synthetic chemicals and antibiotics have low antimicrobial activity or antibiofilm effect against plaque infections and high susceptibility to develop resistance against bacteria. Furthermore, synthetic chemicals possess side effects, which can induce tooth staining, vomiting and diarrhea. It is believed that the uses of medicinal plants are effective and potential substitutes against biofilm forming bacteria and inhibition of dental plaque. The substances which are being used to control dental biofilm should not have any side effects and prevent the attachment of oral pathogen to oral mucosa and teeth [18,19].

A variety of medicinal plants and their extracts are being used for the cure and management of oral diseases [3]. A comprehensive study has estimated the activity of plant products and their extracts for specific oral microbes [20]. Many other studies have suggested that various plants produce anti-biofilm natural phenolic compounds that have a capability to control dental biofilm [21,22]. Gallic acid can prevent the growth of oral microbes and inhibit the dental biofilm formation by *S. mutans* [23,24]. However, for this study different bacteria, including *Proteus* spp., *Escherichia coli*, *Pseudomonas* spp., *Salmonella* spp., *Streptococcus mutans,* and *Staphylococcus aureus* and mixed species bacteria were used.

Phenolic compounds or polyphenols, such as chlorogenic, caffeic, and gallic acids are widely used as a universal group of plant extract, which are highly antimicrobial and have other biological effects [23]. Gallic acid is frequently found in various *Quercus rubor* (oak) bark, *Camellia sinensis* (tea) leaves and seeds of Vitis vinifera (grapes), while caffeic and chlorogenic acids are found in other plants such as Triticum, *Oryza sativa* and *Camellia sinensis* [17,25].

The present study was focused on antimicrobial, biofilm formation and biofilm dispersal efficacy of Gallic acid (GA) against bacteria including *Proteus* spp., *Escherichia coli*, *Pseudomonas* spp., *Salmonella* spp., *Streptococcus mutans*, and *Staphylococcus aureus* and multispecies bacteria.

## 2. Results

### 2.1. Biofilm Forming Potential of Single and Multispecies Bacteria

**Figure 1:**
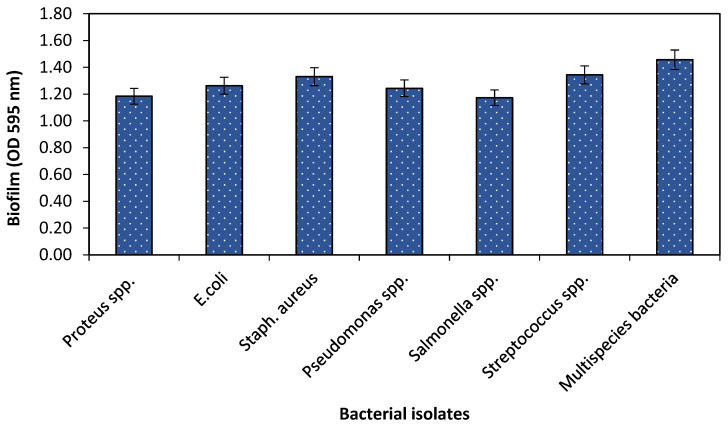
Biofilm forming ability of single and multispecies bacteria.

To evaluate the biofilm formation potential of single and multispecies bacteria, bacteria were grown in 24-well flat bottom polystyrene plates with optical density (OD_600_ 0.001) in nutrient media for 24 h at 37 °C, 120 rpm in a shaker incubator. The plates were then subjected to crystal violet assay after incubation. The biofilm forming ability of single and multispecies bacteria was analyzed by measuring OD at 595 nm in spectrophotometry. The results showed that all bacteria tested have the potential to develop a biofilm. The current study revealed that all bacterial species either single or multispecies, had the ability to form a biofilm. However, multispecies had the most prominent potential of biofilm production as shown in Figure 1.

### 2.2. Antimicrobial Effect of Gallic Acid

**Figure 2:**
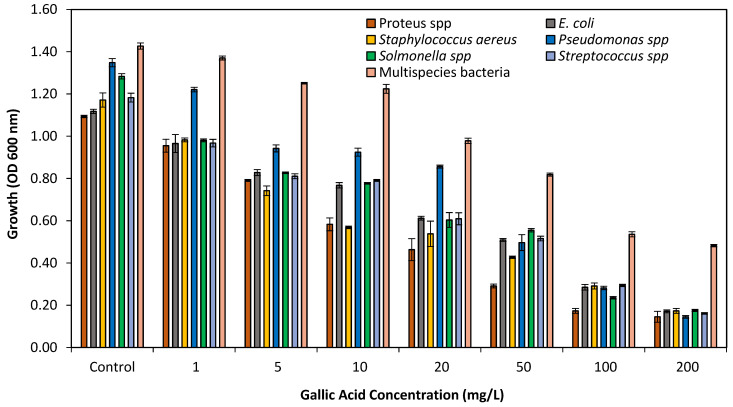
Antimicrobial effects of GA concentrations on growth of single and multispecies bacteria.

The present study showed that at lower concentrations of GA (1–5 mg/L), the growth of bacteria was slightly reduced (12%). Similarly, the bacterial growth (either single or multispecies bacteria) was moderately reduced (58.62%) at 10–50 mg/L concentrations of GA. However, up to 86% growth reduction of single specie bacteria was observed at higher concentrations (100–200 mg/L) of GA and 67% growth reduction was observed against multispecies as shown in Figure 2. The current study revealed that both single and multispecies bacterial growth were reduced at higher concentrations of GA. 

### 2.3. Gallic Acid Effect on Biofilm Formation in Microtiter Plate

**Figure 3:**
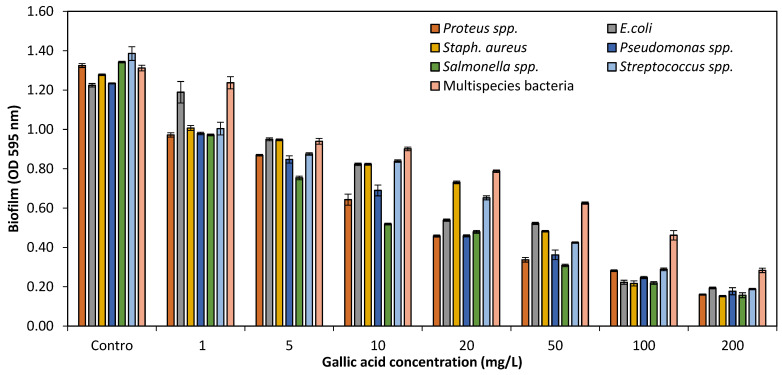
Effects of GA concentrations on biofilm formation of single and multispecies bacteria.

Different concentrations (1–200 mg/L) of GA were tested for their potential effects on biofilm formation against single and multispecies bacteria with OD 0.001 in 24-well polystyrene microtiter plates. Biofilm OD was measured at 595 nm of single and multispecies bacteria. Control (without GA) of all species was also measured. The study indicated that at 1–50 mg/L of GA, up to 66.3% biofilm production against single and multispecies bacteria, and at high concentrations (100–200 mg/L), biofilm formation was significantly reduced up to 85.5%, as shown in Figure 3. The current experimental study showed that GA at lower concentrations (1–50 mg/L) had no notable effects on growth and biofilm formation of single and multispecies. Although, higher concentrations (100–200 mg/L) of GA had prominent inhibitory effects on growth as well as biofilm formation.

### 2.4. Effect of Gallic Acid on Biofilm Dispersal (Multispecies Species)

**Figure 4:**
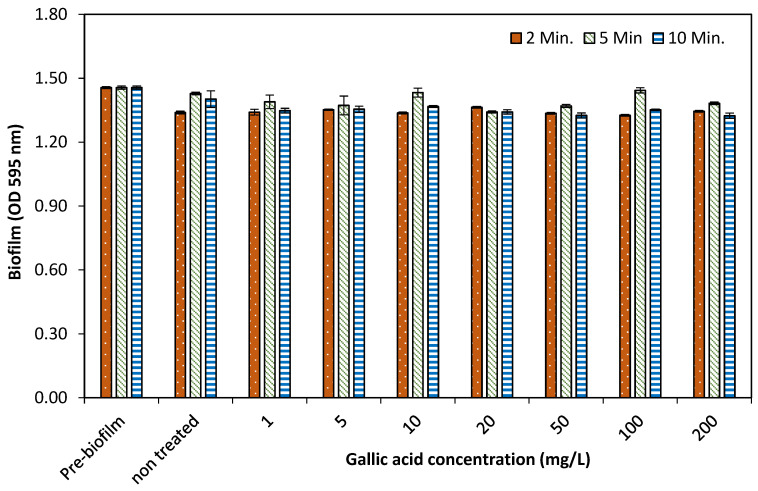
The potential of GA (1–200 mg/L) on the dispersal of 24-h old biofilm of multispecies bacteria treated for different time intervals in the absence of nutrients.

After measuring OD at 595 nm, the biofilm dispersal results showed that all the concentrations of GA used for different time exposures had no obvious dispersal activity, as shown in Figure 4. The current study indicated that GA is not an effective agent for the dispersion of preformed bacterial biofilm under tested conditions used in this experiment.

### 2.5. Effect of Gallic Acid on Bacterial Biomass

**Figure 5:**
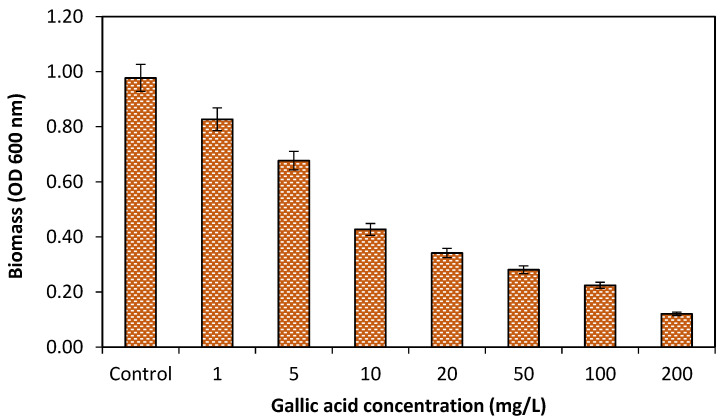
The potential of GA concentrations (1–200 mg/L) on biomass of multispecies bacteria.

**Figure 6:**
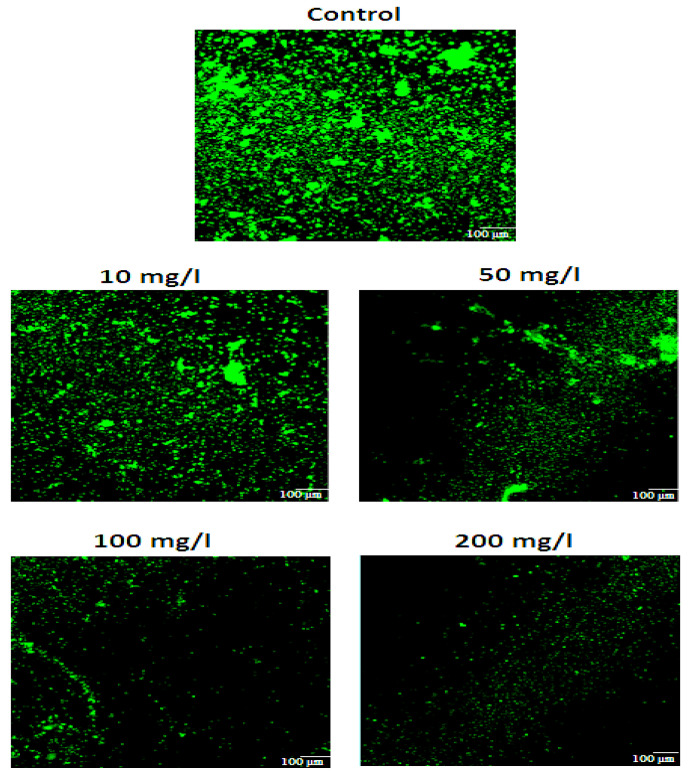
Florescence microscopy images showing stained biofilm cells, scale bars = 100 μm.

All the tested bacteria showed the biomass production in the form of biofilm development on glass surfaces. The production of biomass was potentially reduced by applying different concentrations of GA. Although lower concentrations of GA (1, 5 and 10 mg/L) showed slight biomass reduction (58.19%), while extensive biomass reduction at higher (20 mg/L and above) GA concentrations as compared to the control (without GA). The current study revealed the potential effects of GA on biomass reduction at higher concentrations as shown in Figure 5. Furthermore, the florescence microscopic images showed the biofilm development on treated and control (untreated) glass surfaces, as clearly shown in Figure 6. Images were also processed through BioImageL software for calculation of percent surface coverage and biomass. The surface coverage calculated for control was 30.2%, while it was 12% at 5 mg/L of gallic acid. Furthermore, it was observed that with increasing concentration of gallic acid, biomass surface coverage was reduced to only 2% at 200 mg/L of gallic acid. Moreover, it was observed that 13,612 (µm^2^) biomass was present for the control, while with increasing concentrations of gallic acid, biomass was reduced to 894 (µm^2^) at 200 mg/L of gallic acid Table 1.

### 2.6. Gallic Acid Effects on EPS Production

**Figure 7:**
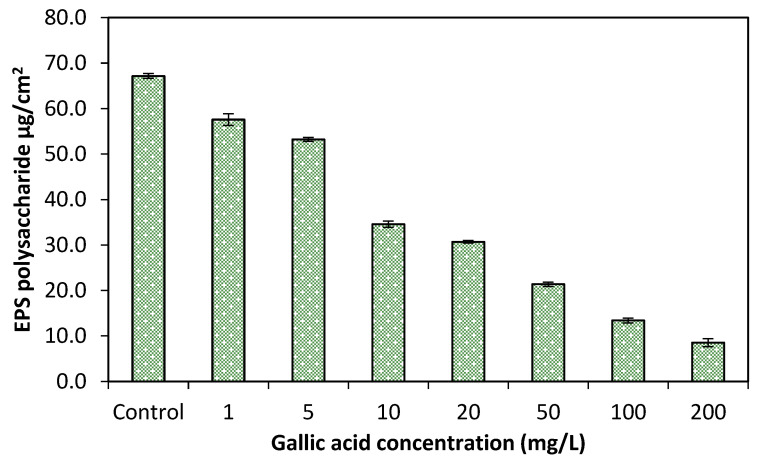
Column chart showing average EPS (polysaccharide) in different concentration (1–200 mg/L) of GA.

For the characterization of biofilm production different concentrations of GA were analyzed to check the effects on extra polymeric substance (EPS). OD was measured at 492 nm and quantified as µg/cm^2^ after the EPS extraction from glass slides. The considerable amount of EPS reduction was observed by applying different concentrations of GA as compared to the untreated sample (control), Figure 7. However, the EPS production was intensively reduced up to 88.6% at higher 20 mg/L and above concentrations of GA than the lower concentrations (1–10 mg/L) < 50%, respectively.

## 3. Discussion

Dental plaque and caries are major oral diseases around the globe and are known to be caused by different microbes and food particles [1]. Many oral bacteria, such as *Streptococcus mutans*, colonize on the teeth surface and are capable of producing acid by fermentation of sucrose and fructose, thereby causing destruction of teeth structure and dental caries [2,3]. Biofilm is the colonization of bacteria entrapped in the exopolymeric matrix formed by the microbial cells. This matrix of biofilm is usually known as glycocalyx, which is typically composed of lipids, lipopolysaccharides [5,6], proteinaceous substances, glycopeptides [7], complex polysaccharide matrix and some other substances that hold together the different microorganisms [8]. 

The current study was mainly focused on control of plaque formation using gallic acid (GA) against six different biofilms forming and cariogenic bacterial strains isolated from dental plaque. Moreover, biofilm forming potential of these bacteria was tested in 24-well polystyrene plates and different concentrations of GA were tested against single and multispecies bacteria to control biofilm development. Further, the dispersal effect of GA on preformed 24-h old biofilm of multispecies bacteria was also evaluated at different time intervals. Furthermore, potential effects of GA on biomass and EPS production by multispecies was estimated on glass slide surface.

The biofilm potential of single and multispecies bacteria was assessed in 24-well polystyrene plates using standard crystal violet staining technique. The results of the present study revealed that the organisms isolated from dental plaque either in single or multispecies bacteria are capable of developing biofilm. A similar study was conducted [26] on four bacterial strains *Listeria monocytogenes, Staphylococcus carnosus, Staphylococcus xylosus* and *E. coli* for their biofilm forming capability using a crystal violet method comparing it with a new method, BioFilm Ring Test^®^. They observed that all microbes had the capability to develop a biofilm and crystal violet method for biofilm detection leading to similar results as the BioFilm Ring Test^®^. Furthermore, the new technique for detection of biofilm was rapid, easier and more reproducible as compared to conventional crystal violet assay. Other groups of researchers have also evaluated the biofilm potential of four bacterial species i.e., *S. aureus, P. aeruginosa, L. monocytogenes* and *E. coli*, and observed same results by developing biofilm in 96-well polystyrene plates [16,17].

Different GA concentrations (1–200 mg/L) were evaluated for the antibacterial effects against single and multispecies bacteria in the present study. Because GA is known to be an antibacterial agent [27], the planktonic growth in nutrient media of single and multispecies bacteria was grown along with desired concentrations of GA was assessed to reveal its effects on bacterial growth. The tested concentrations showed inhibitory effects on bacterial growth, and decreased growth was noticed as with the increasing concentrations of GA. Though, there was no remarkable effect on growth reduction 58.62% at lower concentrations (1–50 mg/L) but at high concentrations (100–200 mg/L) bacterial growth was greatly reduced (67%) against multispecies bacteria and 86% against single species. Another study [28] found that bacterial growth was not greatly affected at concentration lower than (100 mg/L). The results showed that higher concentrations strongly reduced bacterial growth is possibly due to growth inhibitory properties of GA. The study showed that GA can inhibit bacterial growth.

Different concentrations (1–200 mg/L) of GA were tested for its potential effects on biofilm formation against single and multispecies bacteria with OD 0.001 in 24-well polystyrene microtiter plates. Although, all concentrations tested had reduced (66.3%) biofilm formation, but biofilm was not greatly affected at lower concentration (1–50 mg/L) as compared to higher (100 mg/L and above) concentrations of GA, which reduced biofilm formation up to 85.5%. Another researcher [17] also found similar results of two phenolic compounds, ferulic acid (FA) and gallic acid (GA), at concentration (1000 µg/mL) against four biofilm forming bacterial species. The activity was performed to prevent and control the biofilms formed by *Escherichia coli, Staphylococcus aureus, Pseudomonas aeruginosa* and *L. monocytogenes*. The biofilms produced by these bacteria had been inhibited and reduced biofilm formation by both phenolic acids. The ferulic and gallic acid reduced > 70% biofilm production of all bacteria tested. 

The effects of GA on biofilm reduction and prevention could be because of several factors other than antibacterial activity, such as treatment temperature, incubation time and nutrient level, all of which have demonstrated effects on the inhibition activity of GA [29]. The exact mechanism of inhibitory effects of GA on bacterial growth and biofilm development is still not unknown, although some studies have reported that biofilm inhibition may be due to degradation of microbial proteins, cell membrane disruption and enzyme inhibition [30,31,32]. Other reports have suggested that the antibiofilm activity of phenolic compound could be the result of inhibition of quorum sensing (QS) signaling molecules [22,33].

The dispersal effects of different concentrations (1–200 mg/L) of GA against preformed 24 h old biofilms of multispecies bacteria were evaluated under nutrient limited (PBS+GA) condition by treating for different time periods (2, 5 and 10 min). Our results showed that GA have an inhibitory effect on new forming biofilm but revealed no clear dispersal effects on preformed biofilm even at higher concentrations. These results are also supported by another group of researchers who also observed that phenolics have potential inhibitory action on biofilm but showed poor/no dispersal effect [17]. The study observed that the GA can inhibit bacterial growth and biofilm formation but did not disperse or remove preformed biofilm neither in the extracellular matrix nor in the bacteria.

The estimation of the potential effects of GA concentrations (1–200 mg/L) against biomass of multispecies bacteria on glass surface was studied. For the attachment of planktonic cell of multispecies bacteria, the glass slides were placed in Petri dishes. The lower concentrations of GA (1, 5 and 10 mg/L) showed a mild biomass reduction (58.19%). While extensively (93.43%) biomass reduction was observed at higher (20 mg/L and above) of GA concentrations. The current study revealed the potential effects of GA on biomass reduction at higher concentrations. Furthermore, the florescence microscopic images showed the biofilm development on treated and control (untreated) glass surfaces. The surface coverage calculated for control was 30.2%, while it was 12% at 5 mg/L of gallic acid. Furthermore, it was observed that with increasing concentration of gallic acid, biomass surface coverage was reduced to only 2% at 200 mg/L of gallic acid. Moreover, it was observed that 13,612 (µm^2^) biomass was present for the control, while with increasing concentrations of gallic acid, biomass was reduced to 894 (µm^2^) at 200 mg/L of gallic acid.

A group of researchers [17,21] also found similar results with GA and four other different polyphenols, showing reduced biomass of S. mutans. Biofilm inhibitory effects of phenolic acids were tested on biofilm mass and metabolic activity using crystal violet assay and alamar blue assay, respectively. GA showed biomass reduction of *L. monocytogenes* and *E. coli* [17].

To determine whether the GA could reduce the EPS production biofilm were formed on glass slide surface. The results of current study showed that all concentrations of GA have inhibitory effects on EPS production by multispecies bacteria. However, the GA at lower concentrations (1–10 mg/L) had not greatly reduced < 50% EPS production but the higher concentrations (20 mg/L and above) of GA, the EPS production was intensively reduced up to (88.6%). Hence, the study observed positive results for the use of GA to reduce the biofilm formation and EPS, where it is suspected to be the major reason of biofilm development [29].

Since GA can control or inhibit biofilm formation when applied from the start (0 h of incubation), application of GA from the beginning could be more viable. Moreover, GA also showed antibacterial activity against all six types of bacteria and multispecies oral pathogens, which indicate that variety of biofilms formed by bacteria can be controlled. Although, the current study revealed that GA can markedly inhibit and control the biofilm development, we should recognize that the biofilm in the current study was grown on the surfaces of glass slides and polystyrene plates under batch conditions. Therefore, the antibiofilm activity of GA should be confirmed in real conditions or simulated models. This study additionally stresses the capability of phenolic compounds i.e., GA as an emergent source of biofilm controlling agent.

## 4. Materials and Methods

### 4.1. Chemicals and Reagents

Gallic acid, crystal violet stain (powdered), phenol, dimethyl sulfoxide (DMSO) and phosphate-buffered saline (PBS) were purchased from Sigma–Aldrich^®^ (Steinheim, Germany) and culture plates, growth media and polystyrene 24-well microplate were purchased from local market. 

### 4.2. Dental Plaque Bacteria and Culture Conditions

The biofilm sample was collected from a patient by the assistance of an experienced dentist. The dental plaque samples were collected from the surfaces of the teeth and placed in Eppendorf tubes containing 2.0 mL phosphate buffered solution (PBS). Informed consent was obtained from patients in accordance with ethical approval from the ethics committee of Abasyn University. Six different dental plaque bacterial species, including *Proteus* spp., *Escherichia coli*, *Pseudomonas* spp., *Salmonella* spp., *Streptococcus* spp., and *Staphylococcus aureus* as previously isolated and identified were used for the biofilm formation. Heart infusion broth (Oxoid, UK) was used to grow and maintain *Streptococcus spp*., and all other bacterial spp. and maintain in tryptic soya broth and agar (Oxoid, UK). All the bacteria were preserved at 4 °C and by sub-culturing regularly [13,16].

### 4.3. Antimicrobial Assay

Gallic acid (GA), a phenolic compound, was evaluated in the present study for its antimicrobial activity on the growth of single and multispecies bacteria in broth media. Different concentrations of GA (1–200 mg/L) were examined for the inhibition of bacterial growth. An antimicrobial test was performed in 24-well polystyrene plates. Both single and multispecies bacteria were grown in nutrient broth medium at 37 °C for 24 h in a shaker incubator at 120 rpm along with different concentrations of GA. Control was also incorporated in the study without the addition of GA. Bacterial optical density (OD_600_) was measured after 24 h incubation and compared with the control.

### 4.4. Control of Biofilm Formation

Single and multispecies bacteria were grown in 24-well microtiter plates. Plates were labelled for each concentration of GA in triplicate (1–200 mg/L) and three wells were first labelled for control (without GA). Then, 50 µL of bacterial culture was added to all wells to achieve desired concentration of 0.001 OD in each well. Then 50 µL of GA concentrations were added in all wells from sub stock solutions of GA. Then, nutrient media was added to all wells to complete total 1 mL. For Blank (untreated or control) 50 µL of sterilized distilled water was added instead of GA and plates were incubated at 37 °C for 24 h at 120 rpm in shaker incubator. After incubation, the plates were rinsed three times with sterile PBS (pH 7.2). The plates were gently shaken so that non-adherent bacteria were removed, and the remaining bacteria were fixed using 1 mL of 99.9% ethanol for 10 min. The liquid was poured off, and the plates were air-dried. The biofilms were stained by adding 1 mL of crystal violet dye (0.1%, wt/vol, Sigma-Aldrich) for 15 min at room temperature. Tap water was used to rinse off excess stain and it was air-dried. The dye bound to the adherent cells was re-dissolved with 1 mL of 33% (*v*/*v*) acetic acid. It was transferred to cuvette and OD was measured at 595 nm using spectrophotometer [34].

### 4.5. Disruption of Established Biofilm

The dispersal effect of GA was also assessed using pre-formed (24 h old) biofilms by adding different concentrations of GA. Only multispecies bacteria were tested in this experiment in 24-well microtiter plates. Pre-formed biofilms were washed by PBS (pH 7.2). Suitable amounts of GA with sterilized distilled water were added into the wells. First, three wells were labelled as control and no GA was added. Three different treatments were performed in which biofilms were exposed for 2, 5 and 10 min at 30 ± 1 °C in a shaker incubator at 100 rpm. Then the biofilm was measured by the crystal violet assay [35,36].

### 4.6. Petri Dish Biofilm Assays

For this experiment, glass slides were kept in each petri dish and 900 µL of bacterial culture was added to each petri dish and 19 mL of nutrient broth media was to the plate. 100 µL of GA was added from different stock solution to maintain the desired concentration (1–200 mg/L) in the petri dish. Multispecies bacteria were grown on glass surface in petri dish in nutrient broth medium at 50 rpm in shaker incubator at 30 ± 1 °C for 24 h. For control, instead of GA, equal amount of sterilized distilled water was added.

#### 4.6.1. Extraction of Cell Biomass and EPS

After growing biofilms on glass slide surfaces, total biomass, and extracellular polymeric substances (EPS) was extracted using a cell scrapper and it was added to the 5 mL sterilized PBS in the tubes and mixed by vertex mixer for 30 s. And then all tubes were centrifuged using a centrifuge machine at 4 °C for 15 min at 10,000 rpm. The supernatant was considered as soluble EPS and it was poured in a new 10 mL test tube. The pellets in bottom of the tube were regarded as cell biomass. 

#### 4.6.2. Measurement of Cell Biomass Concentration

The pellet at the bottom of the test tubes was washed with the saline water and 5 mL PBS was poured in the tube containing the pellets. It was mixed by vertexing using a vertex machine. Then, OD was determined at 600 nm using a spectrophotometer.

#### 4.6.3. EPS Quantification

Supernatant was considered as soluble EPS and 1 mL was taken from supernatant and poured in a labelled glass tube. Then 0.5 mL of 5% phenol was added in the tube. About 2.5 mL concentrated H_2_SO_4_ solution was added carefully to the mixture. The mixture was incubated for 10 min at room temperature and absorbance was determine using a UV spectrometer at 492 nm [36].

#### 4.6.4. Florescence Microscopy

Biofilm samples on glass were further analyzed by florescence microscope. After incubation, biofilm samples were washed gently with saline water and 0.1% fluorescein isothiocyanate (FITC) was used to stain the biofilm that was then kept in the dark at room temperature for 15 min. To remove unbound stain, the slides were washed with sterilized distilled water. Then, stained slides were subjected to florescence microscopy using Nikon 90i fluorescence microscope (Nikon, Japan). The stained biofilms were visualized, and images were captured at 488 nm excitation and 530 nm emission. Digital images were viewed by NIS-AR Element Software (Nikon, Japan). Images were processed using image analysis software BioImageL^TM^ v.2.1 (Developed by Dr. Luis Chávez de Paz). Percent surface coverage and biomass (µm^2^) was calculated from images using BioImageL^TM^. 

### 4.7. Statistical Analysis

All experiments were carried out in triplicate. Average and standard error was calculated using Microsoft Excel and standard error was presented in the form of error bars in the graphs. Where indicated, a two-tailed Student’s *t* test (*p* < 0.05) was employed for testing the significance of results. This test was performed using Microsoft Excel 2010 (Microsoft Corporation, Redmond, WA, USA).

## 5. Conclusions

From the current research study, it was concluded that all the bacterial species i.e., *Proteus* spp., *Escherichia coli*, *Staphylococcus aureus*, *Pseudomonas* spp., *Salmonella* spp., and *Streptococcus mutans* have the capability to form biofilm. Moreover, different concentrations (1–200 mg/L) of gallic acid (GA) showed antimicrobial effect by reducing growth of single and multispecies bacteria. However, at lower concentrations of GA, bacterial growth was slightly reduced (58.62%) but growth was markedly reduced by 86% by single species and 67% against multispecies at higher concentrations. Furthermore, GA also showed potential effects on biofilm reduction. Biofilm development of single and multispecies bacteria was also markedly (85.5%) controlled at higher concentrations (100 and 200 mg/L) of GA as compared to lower concentrations (66.3%). There was no obvious dispersal effect of GA concentrations observed on preformed 24 h old, preformed biofilm by multispecies bacteria treated for different time intervals (2, 5 and 10 min). Furthermore, potential effects of GA were observed against biomass production by multispecies bacteria grown on glass slides. At higher (20 mg/L and above) concentrations of GA, the biomass was prominently reduced by up to 93.43%. Additionally, EPS (polysaccharide) production was also intensively reduced up to 88.6% at higher (20 mg/L and above) concentrations of GA. 

## Figures and Tables

**Table 1:** Effect of gallic acid on biofilm surface coverage and biomass reduction.

Sample (mg/L)	Surface Coverage (%)	Biomass (µm^2^)	% Biomass Reduction
Control	30.2%	13,612	00.00
10	12%	5691	58.19
50	7%	3169	76.71
100	2.4%	1062	92.19
200	2%	894	93.43
